# Association of Serum Potassium Variability With 60-day Mortality and Cardiovascular Events after COVID-19 Infection in Maintenance Hemodialysis Patients

**DOI:** 10.7150/ijms.88529

**Published:** 2024-01-01

**Authors:** Moxuan Feng, Dan Li, Fengyun Hao, Xin Chu, Lin Li, Qingling Yuan, Haina Li, Yongzheng Hu, Wei Jiang

**Affiliations:** 1 Department of Nephrology, The Affiliated Hospital of Qingdao University, Qingdao, Shandong, China.; 2 Department of Cardiology, The Affiliated Hospital of Qingdao University, Qingdao, Shandong, China.; 3 Department of Pathology, The Affiliated Hospital of Qingdao University, Qingdao, Shandong, China.

**Keywords:** COVID-19, potassium, hemodialysis, mortality, cardiovascular disease

## Abstract

**Objective:** This study aimed to investigate the association between serum potassium variability and 60-day mortality and cardiovascular disease (CVD) in maintenance hemodialysis (MHD) patients following the coronavirus disease 2019 (COVID-19) infection.

**Methods:** We conducted a retrospective study on MHD patients treated at the affiliated hospital of Qingdao University hemodialysis center who were infected with the novel coronavirus between December 1, 2022, and January 31, 2023. Baseline characteristics of patients were collected from electronic medical records. Kaplan-Meier survival analysis was used to obtain patient survival probabilities, and multivariate Cox hazard regression models and binary Logistic regression models were used to obtain hazard ratios (HR), odds ratios (OR), and 95% confidence intervals (95% CI) between exposure and outcomes.

**Results:** A total of 296 patients were included in this study, with a mean age of 57.2±16.3 years, and 59.8% were male. The 60-day mortality rate was 10.8%, and the incidence of CVD was 32.8%. Kaplan-Meier curves showed that a higher potassium variability coefficient was associated with higher all-cause mortality (*P* = 0.024). After adjusting for potential confounders, multivariate Cox regression analysis showed that the HR for 60-day mortality in the Q4 group compared to the Q1 group was 2.06 (95% CI = 1.03-4.09, *P* = 0.040), and binary Logistic regression analysis showed that the OR for 60-day CVD in the Q4 group compared to the Q1 group was 4.09 (95% CI = 1.52-10.97, *P* = 0.005).

**Conclusion:** Increased serum potassium variability in MHD patients after COVID-19 infection significantly increased the likelihood of 60-day mortality and CVD.

## Introduction

The coronavirus disease 2019 (COVID-19) pandemic caused by severe acute respiratory syndrome coronavirus 2 (SARS-CoV-2) has led to unprecedented morbidity and mortality worldwide. Older age, metabolic disorders, lung disease, coronary atherosclerotic heart disease, chronic renal failure, and neoplastic disease are more likely to lead to severe COVID-19 and suggest a poor prognosis^1^. Although electrolyte disturbances can occur in a variety of diseases, in the SARS-CoV-2 pandemic, unpredictable electrolyte disturbances can occur due to respiratory failure and metabolic changes caused by organ failure^2^. Previous studies have shown that the incidence of hypokalemia and hyperkalemia in patients with novel coronavirus pneumonia is 24.3% and 4.15%, respectively 3.

Potassium is one of the main cations in human cells and plays an important role in maintaining cell osmotic pressure and acid-base balance and regulating cardiac and muscle function^4^. The intracellular and extracellular potassium ion gradients are mainly maintained by the Na+/K+-ATP pump, and sudden changes in plasma potassium concentration can cause potential hemodynamic and electrophysiological changes. Potassium homeostasis abnormalities are usually associated with acute kidney injury, chronic kidney disease (CKD), heart failure, and other diseases^5^. In recent years, more and more attention has been paid to the clinical impact of serum potassium variability on patients with various diseases. Studies have found that increased serum potassium variability can lead to a higher risk of all-cause death in maintenance hemodialysis (MHD) patients^6^. Moreover, it has been reported that increased serum potassium variability is associated with a higher risk of cardiovascular events and all-cause mortality in patients with heart failure and CKD^7^.

The MHD patients are a special group of patients with a higher risk of electrolyte disorders. COVID-19 infection may further increase the risk of electrolyte disorders in hemodialysis patients^8^,^9^. However, there is currently limited research on the association between serum potassium variability and the risk of all-cause death and cardiovascular disease (CVD) in MHD patients after COVID-19 infection. Therefore, we conducted this retrospective study to investigate the relationship between serum potassium variability and 60-day mortality and CVD in MHD patients following COVID-19 infection.

## Methods

### 1. Study subjects

This study is a retrospective study of MHD patients infected with COVID-19 between December 1, 2022, and January 31, 2023, at three affiliated hemodialysis centers of Qingdao University. Inclusion criteria: all enrolled patients met the diagnostic criteria for stage 5 CKD, were over 18 years old, had been on dialysis for more than 3 months, and were diagnosed with COVID-19 infection between December 1, 2022, and January 31, 2023. A total of 296 patients were included. The coefficient of variation was used to calculate the variability of serum potassium levels after infection, and patients were divided into four groups (Q1, Q2, Q3, and Q4) according to quartile intervals, with upper quartile, median, and lower quartile values of 0.033, 0.065, and 0.118, respectively. This protocol was approved by the ethics committee of the affiliated hospital of Qingdao University and obtained written informed consent from all participating patients.

### 2. Collection of patient clinical data

General clinical data of enrolled patients were collected from the electronic medical record system, including demographic data [age, gender, dialysis age, body mass index (BMI)] and clinical data (chronic disease history, and laboratory test results). Clinical outcomes were defined as death or CVD within 60 days after COVID-19 infection, with the last day of follow-up being April 1, 2023. CVD diagnostic criteria were based on the WHO MONICA project, and non-CVD death was defined as death unrelated to CVD 10. The occurrence of death and CVD was determined by a panel of physicians, including cardiologists and general practitioners. BMI was calculated as weight in kilograms divided by height in meters squared. Smoking was defined as at least one cigarette per day, hypertension as systolic blood pressure (SBP) ≥140 mmHg and/or diastolic blood pressure (DBP) ≥90 mmHg, or the use of antihypertensive medication within 2 weeks. Diabetes was defined as a fasting blood glucose level of at least 7.0 mmol/L or a previous diagnosis of diabetes. Venous blood samples were collected after at least 8 hours of fasting and stored at -80°C.

### 3. Statistical analysis

All data were analyzed using SPSS (version 26.0) and GraphPad (version 9.0). Continuous normally distributed variables were expressed as mean±standard deviation (mean±SD); non-normally distributed continuous variables were expressed as median and interquartile range; categorical variables were expressed as numbers and percentages. One-way ANOVA, Kruskal-Wallis test, and Chi-square test were used for the significance analysis of the above variables. Kaplan-Meier survival analysis was used to calculate the 60-day mortality, CVD, or non-CVD mortality probabilities for the four groups. The log-rank test was used to analyze the overall survival distribution between groups. Multivariate Cox regression was used to determine the association between serum potassium variability and 60-day all-cause mortality, adjusting for dialysis age, history of diabetes, history of chronic pulmonary diseases, and N-Terminal Pro-Brain Natriuretic Peptide (NT-proBNP). Hazard ratios (HR) and 95% confidence intervals (95% CI) were used to indicate effects. Binary logistic regression was used to determine the association between serum potassium variability and 60-day CVD, with odds ratios (OR) and 95% CI indicating effects. Statistical significance was set at *P* < 0.05.

## Results

### 1. Study Population and Baseline Characteristics

The baseline clinical characteristics of the patients included in the study are shown in Table 1. The average age of the patients was 57.2 (SD 16.3), with 177 males accounting for 59.8% of the total participants. At baseline, 21.3% of the patients had a history of smoking, 87.2% had hypertension, which was the most common comorbidity, 24.2% had diabetes, 6.4% had coronary heart disease, and 9.1% had chronic pulmonary diseases.

Laboratory test results showed that the levels of white blood cells (WBC), neutrophils, and lymphocytes were 6.3 (5.0-7.8) *10^9^/L, 4.3 (3.3-5.8) *10^9^/L, and 1.1 (0.8-1.6) *10^9^/L, respectively. The average level of serum creatinine was 936.0 (687.3-1170.0) μmol/L, NT-proBNP was 5659.0 (2476.0-182770) ng/mL, and C-reactive protein level was 4.6 (1.0-35.0) mg/L.

In this study, patients were divided into four groups based on serum potassium variability levels. Group Q1 included 76 patients with a coefficient of variation less than or equal to 0.033, group Q2 had 73 patients with a coefficient of variation between 0.033 and 0.065, group Q3 had 73 patients with a coefficient of variation between 0.065 and 0.118, and group Q4 had 74 patients with a coefficient of variation between 0.118 and 0.550. There were no statistically significant differences between the four groups in terms of age, gender, BMI, smoking history, hypertension history, and coronary heart disease history.

As serum potassium variability increased, the likelihood of comorbidities also increased. There were significant differences between the four groups in terms of history of chronic pulmonary diseases (*P*=0.028) and diabetes (*P* =0.013), as well as NT-proBNP levels (*P* <0.001). There were no significant differences in other laboratory test results, including WBC, hemoglobin, platelets, neutrophils, lymphocytes, liver function indicators, blood sodium, blood chloride, serum creatinine, blood urea nitrogen, parathyroid hormone (PTH), and C-reactive protein between the four groups (Table 1).

### 2. Association between Potassium variability coefficients and 60-day all-cause mortality and CVD

**Figure 1:**
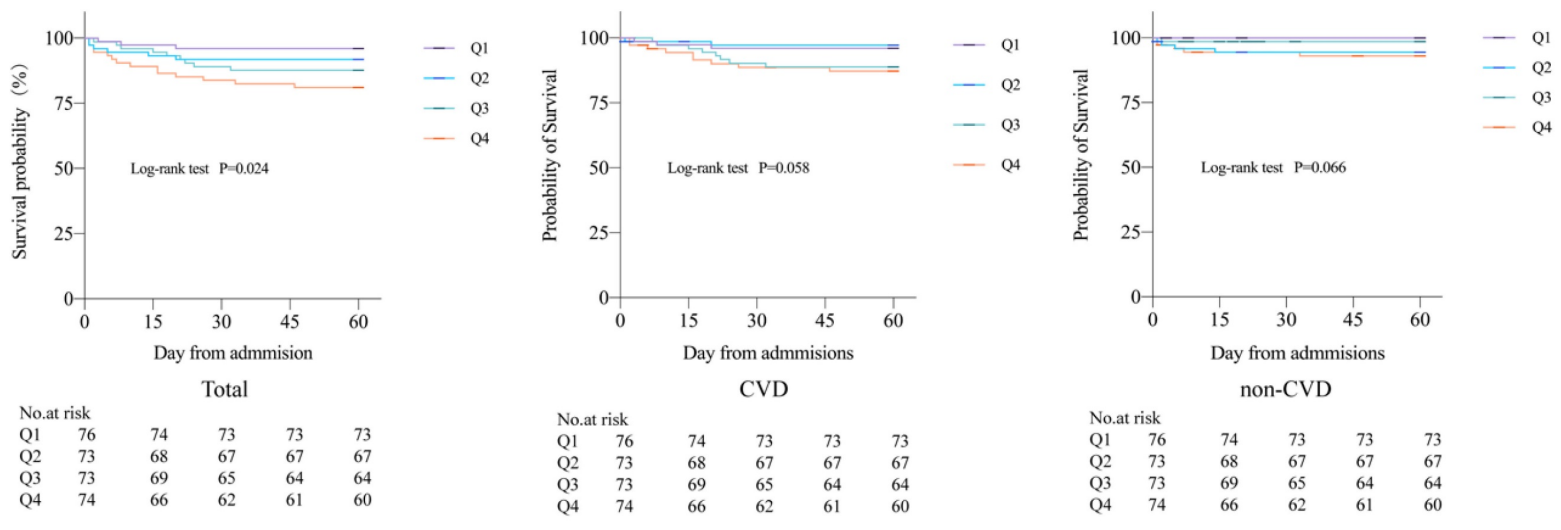
Kaplan Meier curves of 60-day all-cause mortality, CVD mortality, and non-CVD mortality

During the 60-day follow-up period, a total of 32 deaths occurred, accounting for 10.8% of all patients, including 10 CVD deaths (3.4%) and 22 non-CVD deaths (7.4%). A total of 97 cases (32.8%) of combined CVD death and non-fatal CVD were recorded. Kaplan-Meier curves showed all-cause mortality, CVD mortality, and non-CVD mortality rates for the four groups, revealing that patients with higher potassium variability coefficients had increased 60-day all-cause mortality, CVD mortality, and non-CVD mortality rates after COVID-19 infection compared to those with lower coefficients. There were significant differences in 60-day all-cause mortality rates between the four groups (*P*=0.024) (Figure 1).

Compared to group Q1, patients in group Q4 with a variability coefficient had a 60-day all-cause mortality rate of 18.9%, and an HR of 1.73 (95% CI = 1.14-2.62, *P* = 0.010). After adjusting for potential confounding factors in the multivariate Cox regression model, the HR for 60-day all-cause mortality in group Q4 was 2.06 (95% CI = 1.03-4.09, *P* = 0.040) (Table 2). In addition, this study used binary Logistic regression analysis to investigate the association between serum potassium variability levels and CVD in the four groups. The CVD rate in group Q3 was 34.2%, with an OR of 1.68 (95% CI = 0.82-3.44, *P* = 0.157). After adjusting for potential confounding factors, the OR was 3.51 (95% CI = 1.06-11.68, *P* = 0.041). In group Q4, the CVD rate was 41.9%, with an OR of 2.32 (95% CI = 1.15-4.69, *P* = 0.019). After adjusting for potential confounding factors, the OR was 4.09 (95% CI = 1.52-10.97, *P* = 0.005) (Table 3).

## Discussion

SARS-CoV-2 is a highly infectious and pathogenic coronavirus that can cause acute respiratory disease pandemics. As of December 19, 2021, the novel coronavirus pneumonia has infected more than 273 million people and caused over 5.3 million deaths^11^,^12^. With the continuous evolution of the pandemic, the incidence, hospitalization rate, and mortality rate vary among different regional populations. A study in Italy collected data from 20 regions and found an average mortality rate of 7.5%, ranging from 3.1-16.7%, and an average ICU admission rate of 21.4%, ranging from 9.4-45.9% 13. Retrospective studies from China have found that the mortality rate of critically ill COVID-19 patients can reach 38-62% 14,15. Previous studies have shown that factors such as male, older age, comorbidities, medical history, white blood cell count, serum creatinine, C-reactive protein, and erythrocyte sedimentation rate increase the hospitalization and mortality rates of COVID-19 patients 16-20. However, the risk factors for mortality and CVD in MHD patients after COVID-19 infection require further investigation.

Clinically, hypokalemia or hyperkalemia is defined as a serum potassium level below 3.5 mEq/L or above 5.0 mEq/L, respectively^21^. Both conditions are adverse prognostic factors. A prospective study found that elevated serum potassium is an independent predictor of death in patients with severe community-acquired pneumonia^22^. Another study showed that both lower (<3.5 mmol/L) and higher (≥4.5 mmol/L) serum potassium levels are associated with increased mortality risk in patients with acute myocardial infarction^23^. In addition to serum potassium levels, potassium variability has been associated with the progression or death of CVD. Zhang et al.^23^found a statistically significant independent relationship between potassium variability and all-cause in-hospital mortality in patients with acute myocardial infarction. Consistently, another cohort study found that even within the normal range, high potassium variability is an independent risk factor for in-hospital mortality^24^. During the COVID-19 pandemic, more research has focused on the association between serum potassium levels and death or cardiovascular events after infection. A study in China found that compared to patients with serum potassium levels of 4.0-4.5 mmol/L, COVID-19 patients with levels ≥5.0 mmol/L had a significantly increased 30-day all-cause mortality rate^25^. Another multi-provincial cohort study also found that potassium disorders as risk factors are closely related to increased risks of all-cause mortality and cardiovascular events^7^. However, no studies have demonstrated the association between potassium variability and death or cardiovascular events after COVID-19 infection.

MHD is one of the most common and effective treatments for delaying the progression of end-stage renal disease. Studies have reported that the risk of adverse outcomes after COVID-19 infection is higher in MHD patients than in the general population, and end-stage renal disease is also one of the risk factors for severe complications and death from COVID-19^26^-^28^ Potassium imbalance is one of the common complications in MHD patients. In patients with CKD, Luo et al. analyzed 55,266 participants with an estimated glomerular filtration rate (eGFR) <60ml/min·1.73m^2^ and found that CKD patients with serum potassium levels <3.5mmol/L and ≥6mmol/L had more than three times the adjusted mortality rate of the reference group (4.5-4.9mmol/L)^29^. Therefore, maintaining stable blood potassium levels is crucial for MHD patients. However, no studies have yet shown the association between blood potassium variability and clinical outcomes in MHD patients after COVID-19 infection. In terms of clinical treatment, MHD patients currently maintain blood potassium stability mainly by changing dialysis prescriptions and using potassium-lowering drugs, such as sodium zirconium cyclosilicate, during the interdialytic period^30^. Sodium zirconium cyclosilicate is a new, highly selective potassium binder that is currently approved for the treatment of hyperkalemia^31^. In several clinical trials, sodium zirconium cyclosilicate has been found to have stable and effective potassium-lowering effects in both short-term and long-term use and is expected to become one of the main treatments for hyperkalemia in patients with CKD^32^,^33^. In this study, it was found that 20 of the 32 deceased patients (62.5%) did not regularly take sodium zirconium cyclosilicate during the interdialytic period, suggesting that oral potassium-lowering drugs may be one of the protective factors against death or cardiovascular events in patients, but due to difficulties in collecting specific treatment information, the application of potassium treatment drugs was not included in the baseline characteristics.

This study found that the 60-day all-cause mortality rate after COVID-19 infection in MHD patients was 10.8%, and the incidence of cardiovascular events was 32.8%. It also found that patients with higher blood potassium variability had increased 60-day all-cause mortality and cardiovascular event rates. As the blood potassium variability coefficient increased, the HR for all-cause mortality and the OR for the occurrence of cardiovascular events also increased, proving that blood potassium variability can be one of the risk factors for death and cardiovascular events in MHD patients after COVID-19 infection.

The strengths of this study include obtaining at least two blood potassium data points before and after COVID-19 infection in MHD patients, stratifying patients according to blood potassium variability coefficients, and reflecting blood potassium fluctuations after COVID-19 infection. However, this study also has some limitations. First, it is a retrospective study based on a single hospital with three hemodialysis centers and a relatively small number of patients. Second, the time of blood potassium testing after COVID-19 infection was inconsistent among patients, and there was a lack of information on drug treatment to improve potassium imbalance before and after infection, which may have some impact on the study results. However, at least two test results still reflect the acute-phase blood potassium fluctuations after infection.

In summary, the main finding of this study is that blood potassium variability is associated with 60-day all-cause mortality and cardiovascular events in MHD patients after COVID-19 infection. After infection with COVID-19, compared with patients with lower blood potassium variability, the higher the blood potassium variability, the greater the risk of all-cause mortality, and the likelihood of experiencing cardiovascular events also significantly increases. In the future, more stringent control of blood potassium levels and maintaining lower blood potassium variability in MHD patients will be necessary.

## Figures and Tables

**Table 1:** Characteristics of Study Participants by Serum Potassium variability

	Total n=296	Q1(CV≤0.033) n=76	Q2(0.033<CV≤0.065) n=73	Q3(0.065<CV≤0.118) n=73	Q4(0.118<CV≤0.550) n=74	P value
Age (year)	57.2±16.3	53.5±14.2	59.4±20.0	58.8±14.5	57.2±15.5	0.109
Male, n (%)	177 (59.8)	47 (61.8)	42 (57.5)	39 (53.4)	49 (66.2)	0.424
Dialysis age (month)	44.0 (24-80)	36.0(24.0-64.5)	49.0 (24.0-115.0)	48.0 (27.0-79.0)	30.0 (16.8-72.8)	0.008
BMI	24.0±4.3	24.6±4.3	23.9±5.1	24.3±3.8	23.3±4.0	0.312
Smoking, n (%)	63 (21.3)	17 (22.4)	15 (20.5)	16 (21.9)	15 (20.3)	0.868
Hypertension, n (%)	258 (87.2)	63 (82.9)	63 (86.3)	66 (90.4)	66 (89.2)	0.074
Diabetes, n (%)	72 (24.3)	11 (14.5)	14 (19.2)	25 (34.2)	22 (29.7)	0.013
Coronary heart disease, n (%)	78 (26.4)	16 (21.1)	20 (27.4)	21 (28.8)	21 (28.4)	0.265
Chronic pulmonary diseases, n (%)	27 (9.1)	6 (7.9)	4 (5.5)	4 (5.5)	13 (17.6)	0.028
SBP (mmHg)	143.8±16.1	146.1±14.5	141.1±16.9	144.6±15.6	143.3±17.2	0.281
DBP (mmHg)	76.7±9.1	77.7±7.8	75.2±9.8	76.9±9.3	77.0±9.5	0.375
WBC (*10^9/L)	6.3 (5.0-7.8)	6.4 (4.7-7.8)	6.3 (5.1-7.6)	6.2 (5.1-7.7)	6.4 (5.1-8.3)	0.961
Neutrophils (*10^9/L)	4.3 (3.3-5.8)	4.2 (3.0-5.5)	4.2 (3.4-5.5)	4.3 (3.3-5.9)	4.5 (3.3-5.5)	0.852
Lymphocytes (*10^9/L)	1.1 (0.8-1.6)	1.3 (0.8-1.7)	1.2 (0.9-1.6)	1.0 (0.8-1.4)	1.1 (0.7-1.4)	0.237
Hemoglobin (g/L)	106.0 (96.0-116.0)	105.5 (96.5-115.0)	108.0 (97.5-114.0)	105.0 (94.0-116.0)	106.5 (91.5-116.0)	0.851
Platelets (*10^9/L)	190.0 (144.0-243.8)	194.5 (150.3-256.0)	190.0 (154.0-240.5)	178.0 (130.0-234.0)	190.5 (140.0-236.8)	0.419
Sodium (mmol/L)	139.0 (136.8-141.0)	139.9 (137.0-141.3)	139.0 (136.6-141.0)	139.0 (137.0-40.5)	138.7 (136.4-141.7)	0.645
Chloride (mmol/L)	98.6 (96.5-101.0)	98.8 (96.4-101.1)	98.0 (96.9-100.1)	99.3 (96.4-101.8)	98.9 (96.5-101.0)	0.560
Calcium (mg/dL)	2.2 (2.1-2.4)	2.2 (2.0-2.3)	2.2 (2.0-2.3)	2.3 (2.1-2.4)	2.2 (2.1-2.4)	0.741
Phosphorus (mmol/L)	2.0 (1.-2.5)	1.9 (1.6-2.5)	1.9 (1.5-2.5)	2.0 (1.5-2.5)	2.0 (1.5-2.4)	0.979
AST (U/L)	12.4 (10.0-16.0)	12.0 (9.0-16.0)	12.0 (9.0-15.0)	13 (10.1-16.3)	13 (11.0-20.3)	0.118
ALT (U/L)	10.4 (7.0-15.4)	10.0 (7.0-14.0)	11.0 (7.0-16.0)	10.3 (8.0-14.4)	11 (7.0-19.0)	0.350
Albumin (g/L)	38.8 (35.4-41.8)	39.3 (36.0-41.5)	38.8 (35.9-42.3)	39.3 (35.5-42.6)	37.4 (33.9-41.0)	0.365
TBIL (μmol/L)	5.9 (4.4-8.3)	5.5 (3.9-8.7)	5.9 (4.6-7.9)	6.2 (4.3-8.2)	6.2 (4.5-8.6)	0.788
DBIL (μmol/L)	2.2 (1.6-3.3)	2.0 (1.4-2.8)	2.2 (1.5-35)	2.6 (1.7-3.6)	2.3 (1.6-3.2)	0.164
TC (mmol/L)	4.0 (3.2-4.7)	4.2 (3.6-4.7)	3.7 (3.0-4.6)	4.0 (3.3-4.6)	4.0 (3.1-4.8)	0.120
TG (mmol/L)	1.4 (1.0-1.8)	1.4 (1.0-1.7)	1.3 (0.9-1.9)	1.4 (1.0-1.9)	1.3 (0.9-1.9)	0.865
Creatinine (μmol/L)	936.0 (687.3-1170.0)	986.5 (746.8-1216.0)	946.0 (740.1-1187.0)	934 (681.5-1095.0)	871.6 (658.5-1181.7)	0.531
Blood urea nitrogen (mmol/L)	27.4 (21.1-32.8)	26.3 (21.5-31.6)	29.0 (22.4-33.4)	24.9 (20.8-31.1)	26.9 (20.3-34.1)	0.389
PTH (pg/mL)	272.7 (170.7-568.2)	332.8 (182.0-618.3)	297.0 (200.0-573.8)	329 (180.0-522.9)	222.4 (112.5-541.5)	0.139
NT-proBNP (ng/mL)	5659.0 (2476.0-182770)	3533.0 (2190.0-16795.0)	5213.0 (1884.0-22567.0)	6757.0 (3138.5-14461.0)	7743.0 (3827.5-35000.0)	0.000
C-reactive protein (mg/L)	4.6 (1.0-35.0)	1.86 (0.6-12.7)	3.2 (0.6-20.9)	5.5 (1.1-36.4)	9.0 (1.5-62.3)	0.401

Abbreviations: BMI: body mass index; SBP: systolic blood pressure; DBP: diastolic blood pressure; WBC: white blood cell; ALT: alanine aminotransferase; AST: aspartate aminotransferase; TBIL: total bilirubin; DBIL: direct bilirubin; TC: total cholesterol; TG: triglyceride; PTH: parathyroid hormone; NT-pro-BNP: N-Terminal Pro-Brain Natriuretic Peptide.

**Table 2:** Multivariate Cox regression of serum potassium variability with 60-day mortality in MHD patients with COVID-19

Variables	60-day death	Non-adjusted		Adjust*	
	n/N (%)	HR (95% CI)	P-value	HR (95% CI)	P-value
Q1	3/76 (3.9%)	1.0	-	1.0	-
Q2	6/73 (8.2%)	2.14 (0.54,8.57)	0.266	4.31 (0.48,38.58)	0.191
Q3	9/73 (12.3%)	1.79 (0.93,3.45)	0.080	2.55 (0.87,7.44)	0.087
Q4	14/74 (18.9%)	1.73 (1.14,2.62)	0.010*	2.06 (1.03,4.09)	0.040*

* Adjusted for dialysis age, diabetes mellitus, chronic pulmonary disease, and NT-pro-BNP. Abbreviations: HR: hazard ratio; CI: confidence interval.

**Table 3:** Logistic regression of serum potassium variability with 60-day CVD in MHD patients with COVID-19

Variables	60-day CVD	Non-adjusted		Adjust*	
	n/N (%)	OR (95% CI)	P-value	0R (95% CI)	P-value
Q1	18/76 (23.7%)	1.0	-	1.0	-
Q2	23/73 (31.5%)	1.48 (0.72,3.06)	0.286	2.78 (0.98,7.90)	0.055
Q3	25/73 (34.2%)	1.68 (0.82,3.44)	0.157	3.51 (1.05,11.68)	0.041*
Q4	31/74 (41.9%)	2.32 (1.15,4.69)	0.019*	4.09 (1.52,10.97)	0.005*

* Adjusted for dialysis age, diabetes mellitus, chronic pulmonary disease, and NT-pro-BNP. Abbreviations: OR: odds ratio; CI: confidence interval.
